# Comparison of diabetic retinopathy severity grading on ETDRS 7-field versus ultrawide-field assessment

**DOI:** 10.1038/s41433-023-02445-8

**Published:** 2023-05-10

**Authors:** Yamini Attiku, Muneeswar Gupta Nittala, Swetha B. Velaga, Chaithanya Ramachandra, Sandeep Bhat, Kaushal Solanki, Chaitra Jayadev, Netan Choudhry, Samantha Miyoko Ashlyn Orr, Shangjun Jiang, Ye He, SriniVas R. Sadda

**Affiliations:** 1 Doheny Eye Institute, Pasadena, CA USA; 2 Eyenuk, Inc, Woodland Hills, CA USA; 3 Department of Cataract and Refractive Lens Surgery, Narayana Nethralaya, Bengaluru, India; 4 Vitreous Retina Macula Specialists of Toronto, Toronto, Ontario Canada; 5 Octane Imaging Lab, Toronto, Ontario Canada; 6 University of Calgary, Calgary, Alberta Canada; 7 Tianjin Key Laboratory of Retinal Functions and Diseases, Tianjin, China; 8 Tianjin Branch of National Clinical Research Center for Ocular Disease, Tianjin, China; 9 Tianjin Medical University Eye Institute, Tianjin, China; 10 Department of Ophthalmology, University of California Los Angeles, Los Angeles, CA USA

**Keywords:** Retinal diseases, Tomography

## Abstract

**Objectives:**

To compare the diabetic retinopathy (DR) severity level determined when considering only the ETDRS 7-field region versus the entire ultrawidefield (UWF) image.

**Methods:**

In this retrospective, cross-sectional study, UWF pseudocolor images were graded on the Eyenuk image viewing, grading, and annotation platform for the severity of DR considering only the regions within the ETDRS 7-fields as well as the entire UWF image using two different protocols: 1) the simple International Classification of Diabetic Retinopathy (ICDR) scale and 2) the more complex DRCR.net Protocol AA grading scale.

**Results:**

A total of 250 eyes from 157 patients were included in this analysis. Six eyes (2.4%) demonstrated a discrepancy in severity level between the ETDRS 7-field region and the entire UWF image when using the ICDR classification system. The discrepancies were due to the presence of lesions [intraretinal haemorrhage (*n* = 2), neovascular disease (*n* = 4)] in the peripheral fields which were not identified in the ETDRS 7-fields. Fourteen eyes (5.6%) had a discrepancy in severity level between the ETDRS 7-field region and the entire UWF image when using the ETDRS DRSS Protocol AA grading scale. The discrepancies were due to the presence of a higher level of disease [intraretinal haemorrhage (*n* = 4), neovascularization (*n* = 4), preretinal haemorrhage (*n* = 2), scatter laser scars (*n* = 4)] in the peripheral fields.

**Conclusion:**

Although considering regions outside of the ETDRS 7-fields altered the DR severity level assessment in <5% of cases in this cohort, significant and potentially vision-threatening lesions including neovascularization and preretinal haemorrhage were identified in these peripheral regions. This highlights the importance of evaluating the entire UWF region when assessing patients with diabetic retinopathy.

## Introduction

Retinal imaging is an integral part of the classification and management of diabetic retinopathy (DR) both in clinical trials and in real-world practice. The Arlie house classification of DR was the first comprehensive system to classify the severity of DR. The modified Arlie house classification was extended and used in the Early Treatment of Diabetic Retinopathy Study (ETDRS) in the 1990s and is generally considered to be the gold standard for the classification of DR [1]. Later in 2003, the international classification of diabetic retinopathy (ICDR) scale was proposed through a consensus meeting in order to provide a simple and more practical classification system for broad use in clinical environments worldwide [2].

These various classification systems are all based on assessment of retinal pathology located within the ETDRS seven standard fields which consist of seven overlapping 30° retinal fields, which in aggregate include ~75° of the retina. With the development of ultrawide-field (UWF) imaging, a larger field (200°) of the retina can be captured in a single acquisition, thereby allowing visualisation of lesions outside of the ETDRS seven standard field. These peripheral fields could potentially demonstrate more extensive and severe lesions than evident within the ETDRS seven standard fields which may have potential implications for patient management.

In this era of UWF imaging, we need to reconsider the area of the retina evaluated for the classification of DR. In previous studies, ICDR grading of DR on stereoscopic 7-field ETDRS 35-mm colour film slides/images was compared to that on non-simultaneous stereoscopic UWF images [3, 4]. Others have compared diabetic retinopathy severity grading using the ICDR or ETDRS classification system, on UWF images cropped to display the ETDRS 7-field territory compared to that of unmasked UWF images [5, 6]. In our study, we compared the severity level of DR determined when considering only the ETDRS 7-field region versus the entire UWF image on non-stereoscopic UWF images. However, in addition to the ICDR scale, ETDRS DRSS scale from DRCR.net Protocol AA, the current standard for UWF DR assessment, was also used for grading [4].

## Methods

In this retrospective multi-centre cross-sectional study, mydriatic UWF fundus images from diabetic patients presenting to the retina clinic were collected for analysis. All images were captured using the Optos 200Tx fundus camera (Optos plc, Dunfermline, Scotland). Images were transferred to the Doheny Image Reading and Research Lab (DIRRL) for assessment. The study was approved by the institutional review board of University of California Los Angeles. As this was a retrospective study, a waiver of informed consent was granted. The study adhered to the principles of Declaration of Helsinki.

**Fig. 1:**
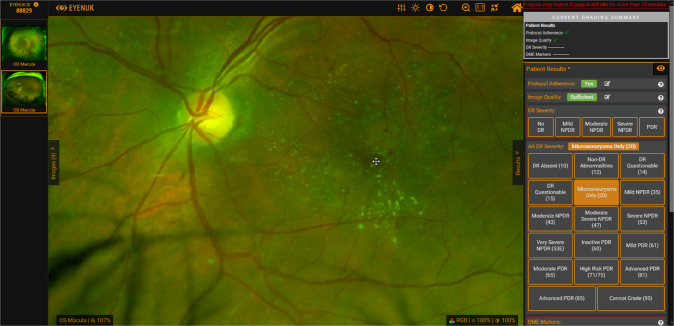
Eyenuk image viewing, grading and annotation platform screenshot. Options available for grading UWF images on ETDRS DRSS Protocol AA classification system is shown.

UWF images were loaded onto the Eyenuk image viewing, grading, and annotation platform (Eyenuk, Inc., Woodland Hills, CA) (shown in Fig. 1) and 250 eyes with DR were randomly selected for assessment in this analysis. Images of poor quality, images with poor capture of peripheral fields and images with artifacts were deemed as ungradable and excluded from the analysis. All images were analysed for the severity of DR by a single certified DIRRL grader (YA). An ETDRS 7-field overlay was superimposed on the UWF images to define the region covered by the ETDRS 7 standard fields. The severity of DR was first graded considering only the region within the ETDRS 7-fields. The severity of DR was then regraded using the entire UWF image. This encompassed the seven standard ETDRS fields and the five extended peripheral fields as described by Silva et al. [7]. Since a single peripheral retinal field encompassed a substantially larger area than a single standard ETDRS field (30°), the severity of lesions in these regions was estimated based on a comparison between the density of lesions within the peripheral field and the density of lesions within the corresponding ETDRS field [6].

While grading the entire UWF image including the peripheral fields, the retinal quadrants (superotemporal, inferotemporal, superonasal and inferonasal) were centred on the optic nerve and extended to the retinal periphery. The DR severity was classified using both the ICDR severity scale and the modified ETDRS DRSS scale from DRCR.net Protocol AA.

In addition to the ETDRS 7-field and UWF retinal assessments, the grader also determined whether the lesions were predominantly central lesions (PCL), predominantly peripheral lesions (PPL) or equivalent using both the single-field method (PPL was assigned if any one peripheral field had more lesions than corresponding ETDRS field, equivalent if there was equal distribution of lesions in each of the quadrants and PCL is none of the peripheral fields had more lesions than the corresponding ETDRS fields) method and the global method (PPL was assigned if the entire region outside ETDRS 7-fields had more lesions than within ETDRS 7-fields, equivalent if the number of lesions within and outside the ETDRS 7-field were equal and PCL if the entire region within the ETDRS 7-fields had more lesions than outside the ETDRS 7-fields), as described in previous reports [7, 8].

## Results

**Fig. 2:**
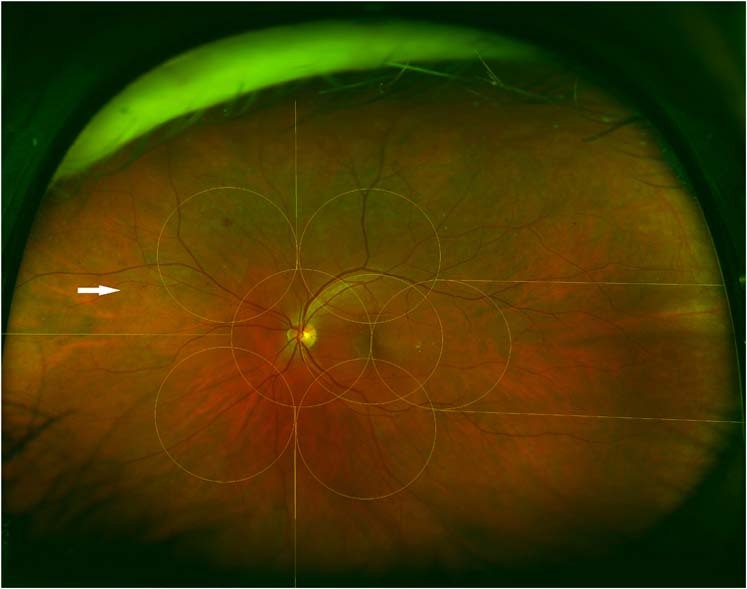
ETDRS 7 -field overlay superimposed on the UWF image. Peripheral field 6 shows neovascularization (arrow) outside the ETDRS 7 fields.

A total of 250 eyes from 157 patients were randomly included in this analysis. When only the seven ETDRS fields were taken into consideration, and the severity of DR was assessed using the ICDR scale, there were 17 eyes with mild NPDR, 157 eyes with moderate NPDR, 30 eyes with severe NPDR and 45 eyes with PDR. When the entire UWF image was considered, 16 eyes were determined to have mild NPDR, 156 eyes with moderate NPDR, 29 eyes with severe NPDR and 49 eyes with PDR [Table 1]. Only 6 (2.4%) eyes demonstrated a difference in DR severity level determined from the ETDRS 7-field region compared to the entire UWF region when using the ICDR classification system. The discrepancy was due to the presence of lesions [higher density of intraretinal haemorrhage (*n* = 2), proliferative disease (*n* = 4)] in the peripheral fields which were not evident in the ETDRS 7-fields [Fig. 2]. The eyes with proliferative disease had flat neovascularization elsewhere (NVE) < ½ disc area without traction in two eyes, flat NVE ≥ ½ disc area without associated traction in one eye and preretinal haemorrhage in one eye in the peripheral fields, in the absence of identifiable proliferative disease in the ETDRS 7-fields. These lesions led to a difference in grading by 1 level in two eyes and by 2 levels in four eyes.

**Table 1:** Distribution of eyes in the different ICDR levels of DR using the ETDRS 7-fields and the UWF images.

ICDR	7 field based (*n*)	UWF based (*n*)
No DR	1	0
Mild NPDR	17	16
Moderate NPDR	157	156
Severe NPDR	30	29
PDR	45	49

The distribution of DR severity levels as determined using the ETDRS DRSS Protocol AA scale is shown in Table 2. Fourteen (5.6%) eyes demonstrated a discrepancy in DR severity level when considering only the ETDRS 7-field region versus the entire UWF region when using the ETDRS DRSS Protocol AA grading scale. The discrepancy was due to the presence of a higher level of disease [increased density of haemorrhage (*n* = 4), neovascularization (*n* = 4), preretinal haemorrhage (*n* = 2), scatter laser scars (*n* = 4)] in the peripheral fields. Of the four eyes with neovascularization in the peripheral fields, two eyes had flat NVE < ½ disc area without traction and one eye had flat NVE ≥ ½ disc area without associated traction, in the absence of identifiable proliferative disease in the ETDRS 7-fields.

**Table 2:** Distribution of eyes in the different ETDRS DRSS levels of DR using the ETDRS 7- fields and the UWF images.

ETDRS DRSS	7 field based (*n*)	UWF based (*n*)
10 - DR absent	1	0
20 - MA’s Only	17	16
35 - Mild NPDR	93	90
43 - Moderate NPDR	39	37
47 - Moderately Severe NPDR	10	10
53 - Severe/ Very severe NPDR	17	16
60 - Inactive PDR	28	32
61 - Mild PDR	12	11
65 - Moderate PDR	21	25
71/75 - High risk PDR	11	12
81/85 - Advanced PDR	1	1
90 - Cannot Grade	0	0

One eye with flat NVE < ½ disc area in the ETDRS 7-fields had additional NVEs in the peripheral fields which were ≥½ disc area and hence fell into a higher severity level while considering the entire UWF region. One eye with no neovascular disease in the ETDRS 7- fields had pre-retinal haemorrhage in the peripheral field and one more eye with only NVE < ½ disc area in the ETDRS 7-fields had preretinal haemorrhage in the peripheral field and thus were graded higher while considering the UWF image. These discrepancies led to a difference in grading by 1 level in six eyes, by 2 levels in two eyes and by more than 2 levels in six eyes.

Seventy-eight eyes (31%) were rated to have PPL and thirteen eyes (5%) had an equivalent distribution of lesions using the single-field method. Thirty-one eyes (12.5%) had PPL and six eyes (2.5%) had an equivalent distribution of lesions using the global method.

## Discussion

In this study, we observed that considering the entire retina rather than just the ETDRS 7 fields can impact the assessment of DR severity. Inclusion of the peripheral field led to the selection of a higher DR severity level in 2.4% and 5.6% of eyes when using the ICDR and ETDRS DRSS Protocol AA classification systems respectively. Importantly, lesions evident only in the peripheral fields that led to a higher classification of DR included the presence of neovascularization elsewhere.

In a prior study, Silva et al. reported a higher severity of DR in 10% of eyes when comparing UWF photographs with ETDRS 7-standard field film photographs [3]. In previous studies comparing peripherally masked UWF images and UWF unmasked images, a discrepancy in DR grading was noted in 8–15% eyes [4–6]. The discrepancy rates from these prior studies are considerably higher than the rate of 5.6% (for ETDRS DRSS) observed in our study. This difference in discrepancy rate between studies may reflect the differences in the distributions of lesions among the cohorts. For example, we observed that only 31% of subjects had predominantly peripheral disease as defined using the single-field method proposed by Silva et al. [7]. This is substantially lower frequency of PPL than reported in prior studies. We observed a similar PPL frequency of 37% in a study of 1406 DR eyes from India [9]. A lower frequency of PPL and peripheral DR lesions overall would presumably decrease the likelihood of detecting lesions in the peripheral fields that would lead to a higher DR severity grade. It is also important to point out that prior studies only compared the 7-field and UWF regions using the modified ETDRS scale. In our study, we also used the common ICDR scale and found an even lower rate of discrepant cases. We did not notice the discrepancy to be affecting a particular quadrant of the retina.

Although less than 6% of eyes demonstrated a higher DR severity when including the peripheral retina in the assessment, it is notable that significant and potentially vision-threatening lesions were detected in the peripheral fields. In particular, six eyes were noted to have evidence of neovascularization in the peripheral retina alone.

Our study does have some limitations that should be considered when evaluating our results. First and most significantly, our study was retrospective and is thus susceptible to ascertainment bias. Thus, the distribution of DR severities and discrepancy rates in our study may not reflect the broader population. Another limitation of our study is that we used only monoscopic images and the ETDRS 7-field assessment was based on effectively cropping the UWF image to only include these regions. It is possible that consideration of stereoscopic images may have impacted the DR severity assessment. Finally, we adapted a previous approach to consider the lesion density across the entire quadrant in order to assess the DR severity in the UWF image. Though it has been used before, the validity of this approach remains uncertain. An UWF-specific grading system may need to be developed in the future as more longitudinal data for eyes with UWF imaging becomes available. Despite the above limitation, a particular strength of our study was the use of certified reading centre DR graders.

In summary, DR severity was rated to be more severe in <6% of DR eyes when the entire UWF image was considered rather than only the region within the 7 ETDRS fields.

Although the number of discrepant cases was low, severe potentially sight-threatening lesions such as neovascularization were missed. This would seem to highlight the importance of assessing the entire retina including the periphery when evaluating eyes with DR.

## Summary

### What was known before

- ETDRS 7 field fundus photos are the gold standard for the diagnosis of diabetic retinopathy.
- Ultrawide field imaging captures about a 200° field of view of the retina.

### What this study adds

- UWF imaging alters the grading of DR in less than 6% of the eyes.
- But potential vision-threatening lesions like neovascularization, preretinal haemorrhage beyond the ETDRS 7-field can be identified on UWF images.
